# Aortic Valve Replacement with Annular Patch Reconstruction for Prosthetic Valve Endocarditis after the Bentall Procedure: A Case Series

**DOI:** 10.70352/scrj.cr.25-0316

**Published:** 2025-08-30

**Authors:** Taiki Matsuoka, Ikuko Shibasaki, Shunsuke Saito, Yusuke Takei, Hirotsugu Fukuda

**Affiliations:** 1 Department of Cardiac and Vascular Surgery, Dokkyo Medical University School of Medicine, Shimotsugagun, Tochigi, Japan; 2 Department of Cardiovascular Surgery, Osaka University Graduate School of Medicine, Suita, Osaka, Japan

**Keywords:** prosthetic valve endocarditis, aortic root replacement, redo aortic valve replacement, Bentall procedure, annular reconstruction

## Abstract

**INTRODUCTION:**

Prosthetic valve endocarditis following aortic root replacement (ARR) typically necessitates redo-ARR, which involves complete graft removal, extensive aortic root dissection, and coronary reimplantation. This highly invasive procedure carries substantial surgical risk, including high operative mortality. In select high-risk patients without evidence of prosthetic graft infection, alternative surgical strategies may reduce procedural complexity and improve outcomes.

**CASE PRESENTATION:**

Here, we report 3 cases of prosthetic valve endocarditis following the Bentall procedure, a common ARR technique, in older patients (mean age: 73.7 ± 3.5 years). All preoperative blood cultures were negative, and no signs of prosthetic graft infection were noted on CT. Due to advanced frailty (Clinical Frailty Scale scores of 7 or 8), conventional redo-ARR was deemed prohibitively high-risk. Risk assessment using the JapanSCORE showed a mean predicted mortality of 32.5% ± 21.0%, with combined mortality and morbidity of 63.7% ± 22.9%. Instead of redo-ARR, annular reconstruction using a bovine pericardial patch was performed, followed by redo aortic valve replacement. All patients underwent successful surgery with no postoperative reinfection. One patient required prolonged intensive care and was transferred to another facility for rehabilitation, while the other 2 recovered uneventfully and were discharged. During a mean follow-up of 26.3 ± 17.6 months, 2 patients died due to non-cardiac causes: one from pneumonia and the other from gastric cancer.

**CONCLUSIONS:**

In high-risk patients without clear evidence of prosthetic graft infection, aortic valve replacement with annular patch reconstruction may represent a viable alternative to redo-ARR, particularly in settings where homografts are not readily available. This approach reduces operative complexity while maintaining structural integrity. Further studies are warranted to validate infection control criteria and assess long-term outcomes.

## Abbreviations

ARR
: aortic root replacement
AVR
: aortic valve replacement
LV
: left ventricular
NCC
: non-coronary cusp
PVE
: prosthetic valve endocarditis
SVG-LAD
: saphenous vein graft-left anterior descending artery

## INTRODUCTION

When PVE occurs after an ARR, redo-ARR is often necessary. Redo-ARR typically requires complete removal of the infected prosthetic valve and graft, followed by ARR. However, this procedure involves extensive aortic root dissection and coronary reimplantation, increasing surgical complexity and risk.^1)^ Redo-ARR is associated with increased morbidity and mortality, owing to preoperative complications, prolonged cardiopulmonary bypass time, and catastrophic reentry injuries.^2)^ Joo et al. reported that the Bentall procedure, a frequently used approach for ARR, is complicated by the risk of infection (in 1.4% of all cases), with most infections occurring within the first 5 years postoperatively.^3)^ In Western countries, homograft root replacement is the preferred treatment strategy to alleviate destructive PVE, as it allows for infection control without introducing additional foreign material.^4)^ However, the use of homografts is limited in Japan due to restricted availability.

To investigate the occurrence of PVE following the Bentall procedure further, we aimed to present 3 high-risk cases in this case series, each with a Clinical Frailty Scale score of 7 or 8 and a mean age of 73.7 ± 3.5 years. These patients showed negative preoperative blood cultures, with no evidence of prosthetic graft infection on CT. Instead of performing redo-ARR, we reconstructed the annular region using a pericardial patch and performed redo AVR.

## CASE PRESENTATION

The requirement of institutional review board approval was waived because the information presented does not include any patient-identifiable information. All study participants provided written informed consent. Notably, intraoperative cultures were not used to guide surgical decisions, as the results were obtained postoperatively. Baseline characteristics (along with preoperative findings) and surgical procedures (along with postoperative outcomes) are summarized in **Tables 1** and **2**, respectively.

**Table 1:** Baseline characteristics and preoperative findings of the 3 cases

Variables	Case 1	Case 2	Case 3
Age (years)	78	71	72
Sex	Female	Male	Male
Time since initial surgery (years)	16	11	1
Initial surgical method	Mechanical Bentall (SJM 21 mm + Hemashield 24 mm)	Bio-Bentall (CEP 25 mm + Triplex 30 mm)	Bio-Bentall (Inspiris RESILIA 27 mm + Gelweave Valsalva 30 mm)
Causative pathogen	*Methicillin-sensitive Staphylococcus aureus*	*Enterococcus faecalis*	*Streptococcus mitis*
WBC on admission (μL)	15500	10200	5700
CRP on admission (mg/dL)	3.41	3.30	7.46
Prosthetic graft infection (preoperative computed tomography)	Negative	Negative	Negative
Preoperative blood cultures	Negative	Negative	Negative
Clinical Frailty Scale Score	8	7	7
Katz Index	0	2	2
Embolism	Negative	Negative	Cerebellar infarction
Vegetation size on transthoracic echocardiography (mm)	8–10	10	10
Preoperative antibiotics	Ceftriaxone Vancomycin Gentamicin	Ampicillin Gentamicin	Ceftriaxone
Antibiotic period (days)	5	17	16
Preoperative WBC (μL)	9400	5400	7800
Preoperative CRP (mg/dL)	7.33	1.5	3.91
Preoperative TTE			
LVEF (%)	29	50	45
JapanSCORE (mortality) (%)	59.0	7.5	31.0
JapanSCORE (mortality and morbidity) (%)	89.5	33.8	67.8

CEP, Carpentier-Edwards Perimount; CRP, C-reactive protein; Inspiris RESILIA, Edwards Lifesciences, Irvine, CA, USA; LVEF, left ventricular ejection fraction; SJM, St. Jude Medical, St. Paul, MN, USA; TTE, transthoracic echocardiography; WBC, white blood cell

**Table 2:** Surgical procedures and postoperative outcomes of the 3 cases

Variables	Case 1	Case 2	Case 3
Surgical procedure	Redo AVR (AVALUS 19 mm), reconstruction of aortic annulus with bovine pericardial patch	Redo AVR (Inspiris RESILIA 23 mm), reconstruction of aortic annulus with bovine pericardial patch	Redo AVR (Inspiris RESILIA 23 mm), reconstruction of partial aortic annulus with bovine pericardial patch
Total operative time (min)	564	400	367
Cardiopulmonary bypass time (min)	310	230	206
Aortic cross-clamp time (min)	217	198	154
Intraoperative cultures^a^	Negative	Negative	Negative
Postoperative antibiotics	Ceftriaxone Vancomycin Gentamicin	Ampicillin Gentamicin	Ceftriaxone
Antibiotic period (days)	50	31	40
Postoperative hospital stay (days)	120	27	40
Hospital transfer	Yes	No	No
Infection recurrence	Negative	Negative	Negative
Postoperative TTE			
LVEF (%)	34	42	32
Aortic valve mean gradient (mmHg)	7	11	9
Postoperative follow-up period (months)	10	45	24
Survival status	Deceased	Alive	Deceased
Cause of death	Pneumonia	–	Stomach cancer

^a^Intraoperative cultures: Culture results are obtained postoperatively and not used to guide intraoperative decision-making.

AVALUS, Medtronic, Minneapolis, MN, USA; AVR, aortic valve replacement; Inspiris RESILIA, Edwards Lifesciences, Irvine, CA, USA; LVEF, left ventricular ejection fraction; TTE, transthoracic echocardiography

### Case 1: A 78-year-old female

#### Surgical details (Fig. 1)

**Fig. 1:**
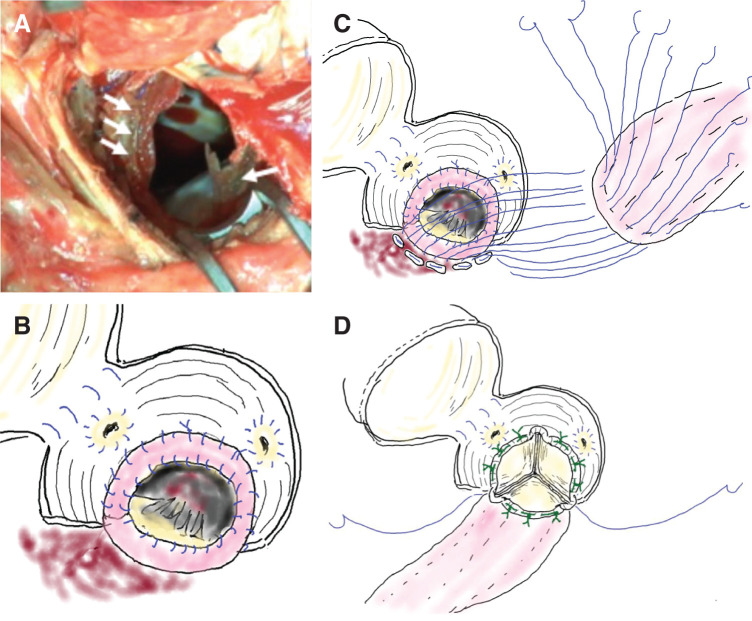
Surgical findings in Case 1 (**A**) Vegetation (indicated with white arrows) is circumferentially attached to the annulus on the left ventricular side. Intraoperatively, circumferential annular tissue destruction is observed. (**B**) After debridement of infected tissue, a bovine pericardial strip is circumferentially sutured to the annulus using 5-0 Prolene, reconstructing the annular structure. (**C**) The aortic defect is repaired with a bovine pericardial patch using a technique similar to annular enlargement. (**D**) Aortic valve replacement is performed using a 19-mm AVALUS prosthetic valve (Medtronic, Minneapolis, MN, USA).

The native ascending aorta showed no signs of tissue fragility or infection. No abscess formation, fistula, or purulent discharge was observed around the prosthetic graft. Aortotomy was performed at the anastomotic site between the native ascending aorta and prosthetic graft.

Vegetation was circumferentially attached to the LV side of the prosthetic valve. After removing the infected valve and tissue, near-complete circumferential annular destruction was noted. To stabilize the anterior mitral leaflet, the NCC portion of the prosthetic graft was incised down to the annular level, and 5 pledgeted 5-0 Prolene mattress sutures were placed.

A bovine pericardial strip (approximately 20 mm in width) was circumferentially sutured to the annulus with 5-0 Prolene to reconstruct the annular structure. A 19-mm AVALUS valve (Medtronic, Minneapolis, MN, USA) was implanted for AVR. The operative time was prolonged due to 2 main reasons. First, adhesions between the prosthetic graft and posterior aspect of the sternum, due to a previous SVG-LAD bypass, resulted in graft injury during redo median sternotomy and required careful management. Second, near-complete circumferential annular destruction necessitated full circumferential reinforcement of the annular defect using a bovine pericardial strip.

### Case 2: A 71-year-old male

#### Surgical details (Fig. 2)

**Fig. 2:**
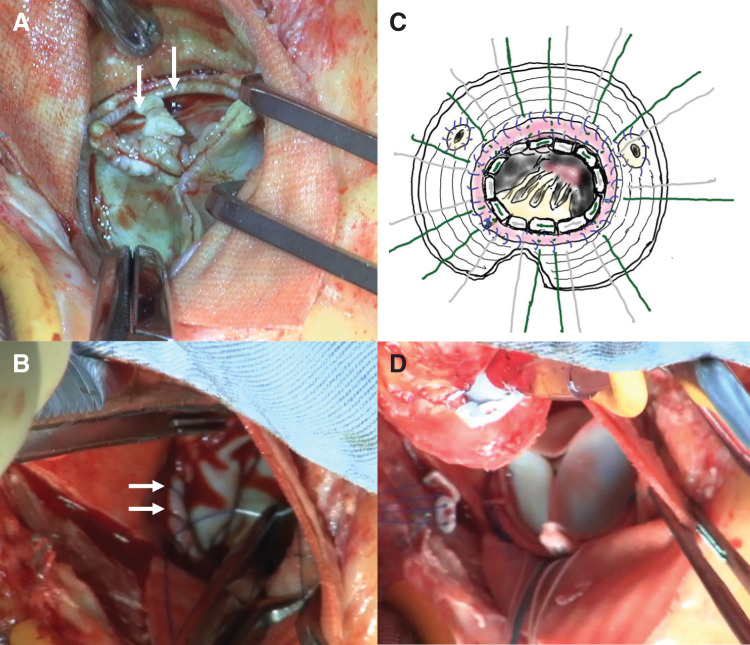
Surgical findings in Case 2 (**A**) Vegetation (indicated with white arrows) is primarily attached to the left coronary cusp region of the prosthetic valve on the aortic side. (**B**) After debridement, a bovine pericardial strip (white arrows) is circumferentially sutured to the annulus using 5-0 Prolene, reconstructing the annular structure. (**C**) Pledgeted 2-0 Ethibond sutures are placed, passing from the left ventricle through the annulus and exiting through the prosthetic graft. (**D**) Aortic valve replacement is performed using a 19-mm AVALUS prosthetic valve (Medtronic, Minneapolis, MN, USA). Valve sutures are passed from the left ventricle through the annulus and secured to the artificial graft.

There were no signs of infection, such as abscess formation, fistula, or purulent discharge, around the prosthetic graft. Transverse aortotomy was performed on the prosthetic graft to gain surgical access. Vegetation was primarily attached to the left coronary cusp region on the aortic side of the prosthetic valve. No annular abscesses were observed. After valve removal, circumferential annular destruction resulted in separation between the LV and the prosthetic graft.

To improve visualization, the NCC portion of the graft was incised down to the annular level. A bovine pericardial strip (approximately 10 mm in width) was circumferentially sutured to the annulus with 5-0 Prolene. A 23-mm Inspiris RESILIA valve (Edwards Lifesciences, Irvine, CA, USA) was implanted. Valve sutures were passed from the LV through the annulus and secured to the prosthetic graft. The graft was then closed and anastomosed. The operative time was prolonged due to near-complete circumferential annular destruction, necessitating full circumferential reinforcement of the annular defect using a bovine pericardial strip.

### Case 3: A 72-year-old male

#### Surgical details (Fig. 3)

**Fig. 3:**
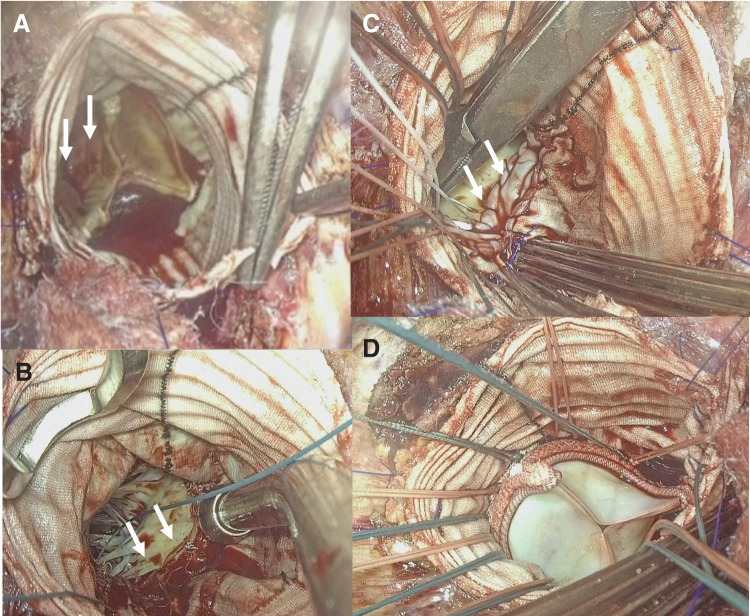
Surgical findings in Case 3 (**A**) Vegetation (indicated with white arrows) is primarily attached to the left coronary cusp region of the prosthetic valve on the aortic side. (**B**) After debridement, partial annular destruction (indicated with white arrows) is observed between the non-coronary cusp region and the mitral valve. (**C**) The defect is closed using a bovine pericardial patch. White arrows indicate the closure site. (**D**) Pledgeted 2-0 Ethibond sutures are placed from the left ventricle through the annulus and prosthetic graft, followed by aortic valve replacement using a 23-mm Inspiris RESILIA valve (Edwards Lifesciences, Irvine, CA, USA).

No signs of infection, such as abscess formation, fistula, or purulent discharge, were noted around the prosthetic graft. The native ascending aorta also appeared free of tissue fragility or infection. Aortotomy was performed at the anastomotic site between the native ascending aorta and the prosthetic graft.

Vegetation was present over all cusps on the aortic side and localized to the NCC region on the LV side. No annular abscesses were found. Upon valve removal, partial annular destruction was limited to the NCC region, causing separation between the LV and the prosthetic graft.

The defect was closed with a bovine pericardial patch (approximately 15 mm in width), allowing partial annular reconstruction. A 23-mm Inspiris RESILIA valve was implanted.

Valve sutures were passed from the LV through the annulus and secured to the graft. The native aorta and prosthetic graft were then anastomosed.

When suturing the patch to the annulus, special care was taken to avoid injury to the conduction system located inferior to the membranous septum. However, in cases where the annular tissue was severely infected or fragile, secure fixation was prioritized over preserving the conduction pathway. In such situations, we proceeded with the understanding that permanent pacemaker implantation might be necessary postoperatively.

## DISCUSSION

To address concerns regarding infection recurrence associated with less invasive surgical strategies for PVE, we established specific indicators to confirm infection control before proceeding with annular reconstruction and valve replacement. These included the following:

(1) administration of appropriate preoperative antibiotics based on identified pathogens or clinical suspicion;
(2) negative preoperative blood cultures;
(3) absence of perigraft low-density areas or intramural gas on contrast-enhanced CT;
(4) advanced age and poor functional status, such as a Clinical Frailty Scale score of ≥7, which implies significantly increased operative risk and limited physiological reserve.

All 3 patients met these criteria, supporting our decision to avoid complete graft removal in selected high-risk cases. None of the patients developed postoperative heart failure, and transthoracic echocardiography before discharge showed an acceptable aortic valve mean gradient. Intraoperative cultures were negative in all cases, and no reinfection occurred during the 10–45-month follow-up.

This approach involves extensive dissection and carries increased surgical risk. Several studies have reported elevated morbidity and mortality associated with redo-ARR due to preoperative complications, prolonged cardiopulmonary bypass time, and risk of catastrophic reentry injuries.^2,5)^

PVE involving the aortic valve is known to be associated with a high operative mortality rate of 24.3% and poor long-term survival (37% at 5 years and 31% at 10 years postoperatively).^6)^ High-risk factors include sepsis, poor LV function, periannular complications, and need for concomitant coronary artery bypass grafting. Aortic root abscesses have been reported in 42% of patients, with *Staphylococcus aureus* infection being a particularly strong predictor of mortality.^6)^

In the 3 cases presented here, the patients had high Clinical Frailty Scale scores (7 or 8), making conventional redo-ARR prohibitively high-risk. Preoperative imaging analysis revealed no evidence of prosthetic graft infection, and subsequent blood cultures returned negative results. Under these conditions, we opted for AVR combined with annular reconstruction using a bovine pericardial patch, thereby avoiding the need for full root replacement. This approach minimized the surgical extent by avoiding root replacement while preserving structural integrity. Although we have no personal experience using prefabricated composite grafts in such cases, we believe that isolated AVR would be structurally infeasible with this type of graft. At our institution, we perform the Bentall procedure by first determining the optimal aortic valve size intraoperatively and then sewing the prosthetic valve to the graft. This technique allows for easier isolated AVR.

Postoperatively, all patients recovered without experiencing reinfection. One patient (Case 1) was transferred to another hospital, while the remaining 2 were discharged. Unfortunately, and subsequently, Case 1 died from pneumonia, and Case 3 succumbed to stomach cancer; however, both deaths were not related to recurrent PVE. These outcomes suggest that AVR with annular reconstruction may serve as a feasible alternative to redo-ARR by avoiding root replacement in carefully selected high-risk patients. The extent of annular destruction was categorized and described for each case in the Case Presentation section to improve reproducibility, given that photographic documentation was limited by intraoperative conditions.

Pocar et al.^4)^ reported favorable outcomes when using homograft root replacement for PVE. The use of homografts enables treatment of destructive infections without implanting additional foreign material and avoids the risks associated with rigid prosthetic devices, such as dehiscence in fragile, infected tissues. In their series, the operative mortality rate was 13%, with 1- and 3-year survival rates of 75.2% ± 5.6% and 70.0% ± 6.3%, respectively. Predictive factors for operative mortality and perioperative low cardiac output included elevated baseline aspartate transaminase and combined mitral valve procedures.

While homografts are widely used in Western countries, their availability in Japan is limited. Therefore, our approach of AVR with annular patch reconstruction using bovine pericardium offers a practical and valuable alternative in the Japanese clinical setting. This technique reduces the surgical complexity while preserving anatomical and functional outcomes and may be particularly useful in managing high-risk patients with destructive PVE in whom homografts are not an option.

## CONCLUSIONS

In high-risk patients without clear evidence of prosthetic graft infection, AVR with annular patch reconstruction may represent a viable alternative to redo root replacement, particularly in settings where homografts are not readily available.

This approach avoids root replacement while maintaining acceptable surgical risk and achieving favorable short- to mid-term outcomes. Further studies are warranted to validate the infection control criteria used in this study, including the role of intraoperative rapid cultures.

## DECLARATIONS

### Availability of data and materials

The data underlying this article are available within the article.
